# Impact of BMI, heart rhythm, and heart rate on photon-counting detector virtual coronary calcium scoring

**DOI:** 10.1007/s10554-025-03419-5

**Published:** 2025-05-19

**Authors:** Judith Becker, Josua A. Decker, Stefanie Bette, Franziska Braun, Luca Canalini, Claudia Wollny, Christian Scheurig-Muenkler, Thomas Kroencke, Florian Schwarz, Franka Risch

**Affiliations:** 1 Department of Diagnostic and Interventional Radiology, University Hospital Augsburg, Stenglinstr. 2, 86156 Augsburg, Germany; 2 Centre for Advanced Analytics and Predictive Sciences, University of Augsburg, Universitätsstr. 2, 86159 Augsburg, Germany; 3 Clinic for Diagnostic and Interventional Radiology, Donau-Isar-Klinikum, Perlasberger Straße 41, 94469 Deggendorf, Germany

**Keywords:** Coronary Artery Calcium Quantification, Photon-counting detector computed tomography, Angiography, Virtual non-contrast, Body mass index, Heart rhythm and rate

## Abstract

The study evaluates the impact of body mass index (BMI), heart rate and rhythm on coronary artery calcium scoring (CACS) derived from calcium-sensitive virtual non-contrast (VNC) series of photon-counting detector (PCD) computed tomography angiography (CTA) compared to true non-contrast (TNC) series. Patients who underwent cardiac imaging with TNC and CTA on a PCD-CT were included. Agatston scores from TNC and VNC images were used to assign CACS risk category. Analyses considered BMI, heart rhythm and heart rate. Distributions were tested for differences between TNC and VNC derived scores and their correlation was assessed. The final cohort included 88 patients. CACS on VNC showed an underestimation of TNC derived values on median Agatston score TNC = 542 (IQR 200–1294), on median Agatston score VNC = 449 (IQR 130–1183), p < 0.001, percentage difference − 11%. However, linear correlation coefficient was high (r^2^ = 0.95), and the CAC severity was categorized equivalent in 80%. In approximately 11% of the study cohort, the misclassification of CAC severity could have potentially led to inappropriate treatment following established guidelines. An impact on the significance and extent of the difference in CACS for BMI > 28 kg/m^2^ and heart rate groups > 69 bpm was found. VNC reconstructions from PCD-CT reliably estimates TNC CACS for BMI ≤ 28 kg/m^2^ and heart rate ≤ 69 bpm in patients with severe coronary artery disease. Potential underestimation of risk category, especially with increased BMI and heart rate, must be considered for clinical decision making.

**Supplementary Information:**

The online version contains supplementary material available at 10.1007/s10554-025-03419-5.

## Introduction

Coronary computed tomography angiography (CTA) is becoming increasingly important in medical diagnosis, with both coronary CTA and coronary artery calcium scoring (CACS) now established as non-invasive diagnostic tools for the diagnosis of coronary artery disease (CAD), risk stratification and assessment of major adverse cardiac events [1–5]. CACS is performed on true non-contrast (TNC) series and allows categorization into no, mild, moderate and severe risk calcification with a high prognostic value regarding the clinical outcome [3]. An increasing number of studies have shown that virtual non-contrast (VNC) reconstructions, which virtually subtract the iodinated contrast agent from spectral CTA studies by means of material differentiation, are also suitable for CACS and promise to reduce radiation dose and examination time by eliminating the need for a separate unenhanced scan [6–9]. However, studies criticized the accuracy of CT values depending on the patient's body mass index (BMI), heart rate, and degree of coronary sclerosis [10–12]. A high BMI necessitates the use of increased radiation doses and amplifies image noise, which diminishes overall image quality. Elevated heart rates are particularly prone to motion artifacts, while non-sinus rhythms complicate synchronization, further degrading image quality. As post-processing algorithms, such as the generation of VNC image series, are very sensitive to statistical inaccuracies in input data, an unacceptable gain in noise can result [13]. Thus, a high image quality is crucial for achieving accurate VNC reconstructions. Poor image quality may impair the precise detection and quantification of coronary artery calcium, potentially resulting in underestimation or misclassification. Since photon-counting detector CT (PCD-CT) scanners have been introduced, the inherently acquired spectral data allow for the routinely reconstruction of VNC images providing a high diagnostic utility [14–17]. Next to the conventional VNC algorithm, a novel calcium sensitive algorithm, namely PureCalcium, has been made available. This algorithm creates a mask of calcium containing voxels prior to the conventional material decomposition into iodine and water, which is then used to restore the original calcium contrast. In terms of CAC quantification, higher calcium scores can be measured that do not necessarily require a conversion factor to approximate scores derived from TNC series, as is usual with conventional VNC [16]. In the following, the term VNC is used to refer to the calcium-sensitive algorithm, as distinct from the conventional one.

Nevertheless, the reproducibility and reliability of CAC quantification on VNC images derived from PCD-CT with respect to patient characteristics remains unclear. The aim of this study was to investigate the influence of BMI, heart rhythm and heart rate on the reliability of VNC series-based calcium scores compared to TNC series as ground truth.

## Materials and methods

### Patients

This single-center study is in line with the principles of the Declaration of Helsinki and was approved by the institutional review board (Ludwig-Maximilians-University Munich, clinical trials NCT04996693). Written informed consent was obtained from all individual participants included in the study. The clinical trial includes “People 18 years of age or older referred for an unenhanced CT or contrast-enhanced CT of the chest/thorax confirmed by a board-certified radiologist” and patients were prospectively enrolled for CACS evaluation. We retrospectively examined the influence of BMI, heart rhythm and heart rate. For our study cohort, consecutive patients with a PCD-CT scan of the heart as part of the preliminary examination to transcatheter aortic valve replacement (TAVR) between January 2022 and March 2023 were considered. This cohort of patients was chosen because they are usually examined uniformly and without medication (e.g. beta-blockers) and often have concomitant CAD. Inclusion criteria were (1) completeness of scan protocol and consistency of scan settings; (2) availability of raw data for uniform image reconstruction; (3) no presence of coronary stents or bypasses. Patient characteristics including sex, age, and BMI were obtained from electronic medical records. Patients were categorized by BMI into normal weight, with a BMI less than 24 kg/m^2^ (BMI_<24_), overweight with a BMI between 24 and 28 kg/m^2^ (BMI_24−28_) and obesity with a BMI greater than 28 kg/m^2^ (BMI_>28_) [18–20]. Regarding heart rhythm, no sinus (HRh_no_sin_) and sinus rhythm (HRh_sin_) were differentiated. Patients' heart rates were categorized from 60 to 90 bpm in 10 bpm increments, HR_<60_, HR_60-69_, HR_70-79_, HR_80−89_ and HR_>89_.

### CT protocol

Scans were performed on a dual-source PCD-CT (NAEOTOM Alpha, Siemens Healthcare GmbH, Erlangen, Germany), including an unenhanced scan and a CTA of the heart. For this study only spiral acquisitions with a high pitch factor for TNC and a low pitch factor for CTA and a constant tube voltage of 120 kVp were considered with a scan range covering the heart. The CTA was electrocardiographically triggered. Reference tube current time product was adjusted by setting the image quality level to 19 for TNC and 50 for CTA. For the readout of spectral information, the dedicated acquisition mode Quantum Plus (Siemens Healthcare GmbH, Erlangen, Germany, with the following detector-based energy thresholds: 20, 35, 65 and 70 keV) was used. Collimation was 144 × 0.4 mm. Heart rhythm and heart rate were obtained from the automatically generated electrocardiograms. As no beta-blockers were administered, only minor differences between scans were expected, and statistical analyses refer to the heart rhythm and heart rate measured during the TNC scan.

For the CTA a triphasic contrast injection protocol with bolus tracking was used. In the first phase 60 ml of undiluted contrast material (Ultravist, Iopromid 300 mgI/ml, Bayer Vital GmbH, Leverkusen, Germany) was injected followed by a mixture of 30 ml contrast material and 30 ml normal saline solution and finalized with 20 ml saline solution. A flow of 5 ml/s was used in all three phases.

Dose information, including dose length product (DLP), volumetric computed tomography dose index (CTDI_vol_), and size-specific dose estimate (SSDE) were extracted from the automatically generated structured dose report.

### Image reconstruction

All reconstructions were performed on the scanner console using the quantitative regular kernel (Qr36) optimized for quantitative analyses and spectral postprocessing with the quantum iterative reconstruction algorithm at strength three. TNC images were generated from the unenhanced scan, and VNC images from the CTA using an iodine subtracting and calcium preserving VNC postprocessing algorithm, both at a virtual monoenergetic image impression of 70 keV. Slice thickness and increment were consistently 3.0 mm and 1.5 mm. Field of view with a matrix size of 512 pixels and number of slices were adjusted to cover the whole heart.

### Image analysis

Noise analyses were performed using Python (version 3.9). As a measure of quantitative image quality, the global noise level was calculated. Of each patient and reconstruction three slices, approximately equidistant to each other and the scan range margins, were selected and their noise map generated. As described previously by Christianson et al., a noise map consists of the standard deviation of CT values for each pixel within one image calculated using a filter of 6 mm size [21]. The histogram of the noise map reveals the most frequent standard deviation of CT values within the respective slice. The average of the most frequent standard deviation of the three slices was taken as global noise level representing the whole image volume (Supplemental Fig. 1).

**Fig. 1:**
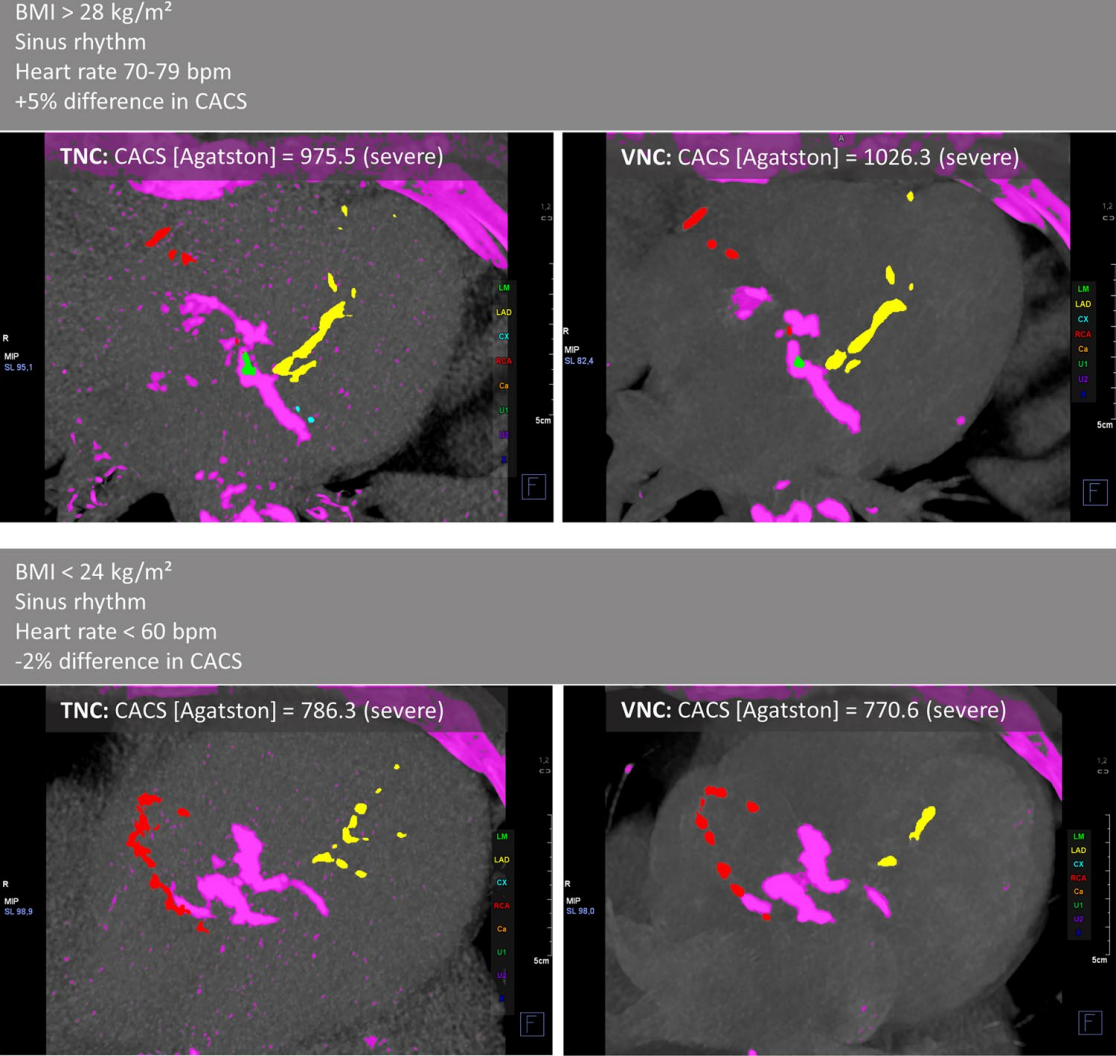
Demonstration of coronary artery calcium scoring (CACS) for two examples. Images show maximum intensity projections of axial slices for true non-contrast (TNC) and virtual non-contrast (VNC) reconstructions. Voxels with CT-values exceeding 130 HU are considered to represent calcifications (marked purple) and can be allocated semi-manually to single coronary arteries: green = left main artery (LM), yellow = left anterior descending artery (LAD), blue = circumflex artery (CX), red = right coronary artery (RCA). *BMI* body mass index

Calcium quantities were determined using commercially available software on a dedicated workstation (Syngo.via, version VB60A, Siemens Healthcare GmbH, Erlangen, Germany). Contiguous voxels with an attenuation above a threshold of 130 HU were detected and semi-manually assigned to the respective coronary artery. The Agatston score was quantitatively exported on a per-patient level (Fig. 1).

### Statistical analysis

Statistical analyses were performed using Python (version 3.9). All data were tested for normal distribution using the Shapiro–Wilk test. Continuous parametric data are presented as mean ± standard deviation, nonparametric data as median with interquartile range, and binary data as frequencies with proportions. Differences between TNC and VNC distributions were tested for their significance using the t-test or Wilcoxon signed-rank test for parametric and nonparametric data, respectively. P-values of multiple comparisons (such as within the groups of BMI, heart rhythm and rate) were corrected with Bonferroni method and considered to indicate statistically significant differences if  ≤ 0.05. Percentage difference was calculated as (VNC – TNC) / TNC * 100%. For linear regression analyses, data was square root transformed to approximate normal distribution and to improve homoscedasticity. To evaluate the linear model’s predictive value, the coefficient of determination (r^2^) was calculated. CAC risk category agreement between TNC and VNC was calculated using the categorization into no, mild, moderate, and severe calcification with an Agatston score of 0, 1–100, 101–400 and > 400.

## Results

### Patient baseline characteristics

**Fig. 2:**
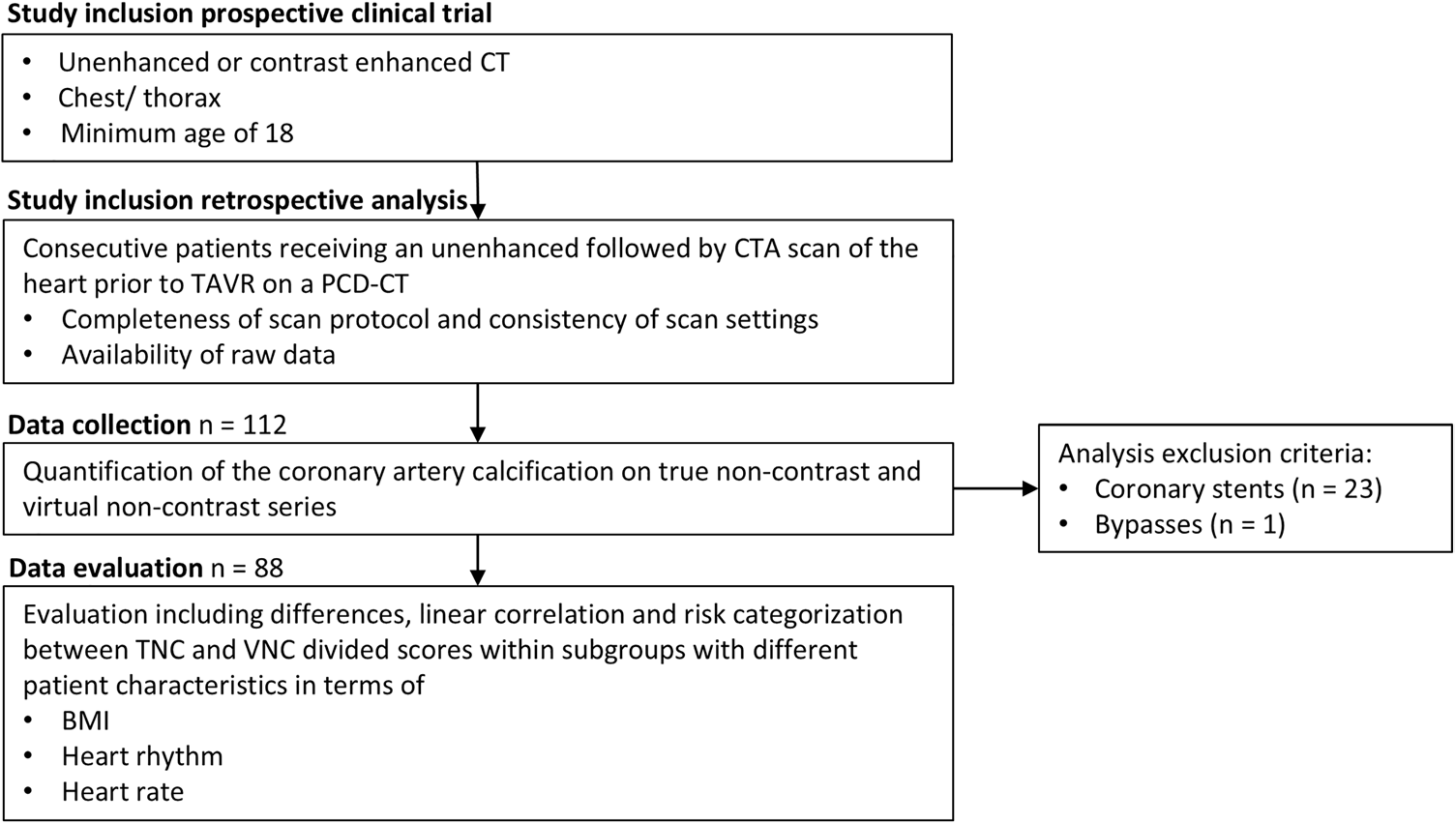
Flowchart demonstrating study inclusion, data collection and evaluation criteria. *BMI* body mass index, *CTA* computed tomography angiography, *PCD-CT* photon-counting detector CT, *TAVR* transcatheter aortic valve replacement, *TNC* true non-contrast, *VNC* virtual non-contrast

In total, 112 patients were enrolled in this study. Thereof, 24 were excluded due to coronary stents (n = 23) or bypass (n = 1) (Fig. 2). The final study cohort consisted of 88 patients, 52 women and 36 men, with a mean age of 79 years. Table 1 lists all values concerning clinical parameters, scan protocol and dose parameters, as well as CAC categories. Defining the total radiation exposure as sum of the unenhanced and CTA scan, the share of the unenhanced scan is about 6% (median proportions CTDI_vol_ = 5.1%, DLP = 6.2%, SSDE = 5.0%).

**Table 1:** Study baseline characteristics including clinical and CT protocol and radiation dose parameters for the unenhanced scan and the angiography

Total n = 88		
Clinical		
Age [years]	78.9 ± 6.1	
Female	52 (59%)	
BMI [kg/m^2^]	27.1 ± 5.1	
Sinus rhythm	62 (70.5%)	
Heart rate (during TNC acquisition) [bpm]	75 (62.8–86.3)	
CT protocol	Unenhanced	CTA
Image quality level	19	50
Pitch factor	3.2	0.21 (0.17–0.24)
CT radiation dose		
Mean CTDI_vol_ [mGy]	1.4 (1.1–1.7)	28.3 (18.7–38.3)
DLP [mGy*cm]	27.9 (22.6–34.3)	437.5 (299.5–666.3)
SSDE [mGy]	1.8 (1.6–2.1)	36.4 (27.6–50.5)
Coronary artery calcification	TNC	VNC
Total score [Agatston]	541.7 (200.2–1293.9)	449.3 (129.6–1182.5)

According to their distribution, values are mean ± standard deviation (age, BMI); median (interquartile range: Heart rate, Pitch factor, mean CTDI_vo_, DLP, SSDE, Total score) or frequency (percentage: Female, Sinus rhythm). *BMI* body mass index, *CTA* computed tomography angiography, *CTDI*_*vol*_ computed tomography dose index, *DLP* dose length product, *image quality level* measure of reference tube current time product, *SSDE* size-specific dose estimate, *TNC* true non-contrast, *VNC* pure calcium virtual non-contrast

### Image noise

The global noise level on TNC series with an average of 22 ± 4 HU was significantly (p < 0.001) higher compared with VNC series with an average of 10 ± 2 HU.

### Calcium scoring

**Fig. 3:**
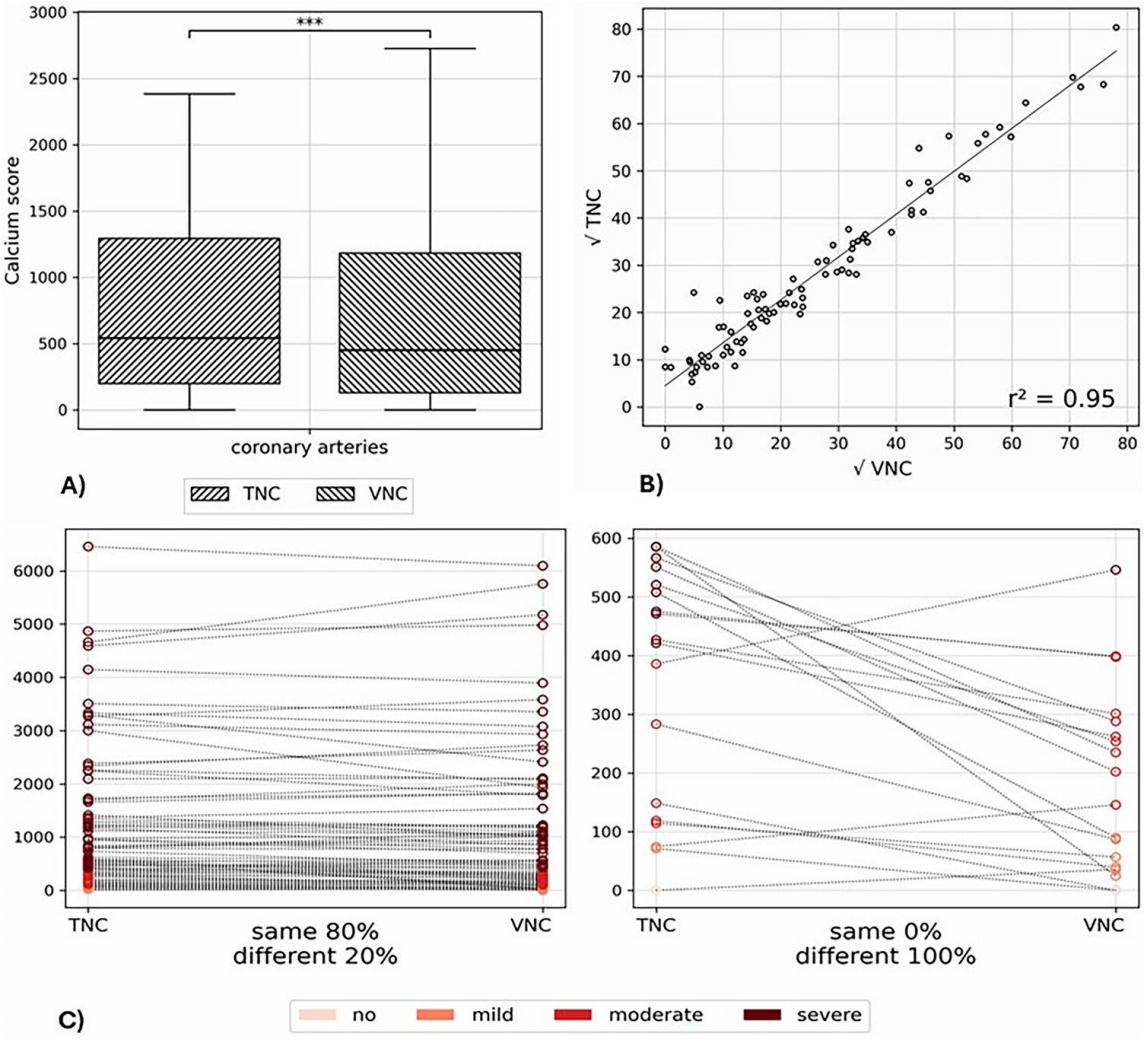
Comparison of the total calcium scores derived from true non-contrast (TNC) and virtual non-contrast (VNC) reconstructions. In **A** the absolute measurements are compared in a box plot (*** = p < 0.001), in **B** the linear regression of the square root transformed values are demonstrated (r^2^ = coefficient of determination) and in **C** the agreement in risk categorization is shown for all patients on the left and only for the misclassified patients on the right

In Table 2 total and subgroup results are listed. Overall, CAC scores measured on VNC differed from TNC-based scores (see Fig. 3A) by a median of − 11%. However, there was excellent linear correlation (r^2^ = 0.95) (see Fig. 3B) and 80% agreement in risk categorization (see Fig. 3C).

**Table 2:** Coronary artery calcification derived from true non-contrast vs. derived from virtual non-contrast grouped by body mass index, heart rhythm and heart rate

Group		n	Absolute CAC			Difference	Linear correlation	Category agreement
			TNC	VNC	p-value	(VNC-TNC)/TNC	r^2^	TNC = VNC %
Total		88	542 (200–1294)	449 (130–1183)	< 0.001	− 11 (− 36 – 2)%	0.95	80
BMI [kg/m^2^]	< 24	23	400 (172–1224)	354 (141–1144)	0.5	− 10 (− 25 – 4)%	0.97	87
	24–28	30	603 (290–1713)	563 (195–1819)	0.2	− 8 (− 28 – 9)%	0.93	77
	> 28	30	659 (362–1238)	476 (140–1083)	< 0.001	− 20 (− 47– − 6)%	0.96	83
Heart rhythm	No sinus	26	700 (354–2098)	633 (234–1820)	< 0.05	− 13 (− 29 – 8)%	0.91	81
	Sinus	62	477 (138–1194)	399 (88–1086)	< 0.01	− 12 (− 48 – 1)%	0.96	79
Heart rate [bpm]	< 60	12	888 (732–1254)	971 (650–1305)	0.3	− 5 (− 19 – 10)%	0.93	83
	60–69	22	601 (361–1723)	552 (266–1953)	0.8	− 6 (− 21 – 9)%	0.98	77
	70–79	19	471 (166–1226)	301 (160–1101)	< 0.05	− 15 (− 40– − 5)%	0.94	63
	80–89	18	395 (107–1088)	167 (63–1005)	< 0.05	− 26 (− 54– − 5)%	0.95	89
	> 89	17	532 (190–1232)	491 (102–1008)	< 0.01	− 26 (− 33– − 6)%	0.91	88

Columns include the number of patients, the absolute measured calcification in each series and the p-value of their distribution differences, their percentage difference, the coefficient of determination of their linear correlation, and their agreement in risk category

Values are median (interquartile range) according to their distribution. *CAC* coronary artery calcium, *BMI* body mass index, *n* absolute number of patients, *r*^2^ coefficient of determination, *TNC* true non-contrast, *VNC* pure calcium virtual non-contrast

The BMI groups contained 23, 30 and 30 patients for BMI_<24_, BMI_24−28_ and BMI_>28_ respectively. Due to missing weight or height information, 5 patients were not considered for BMI subgroup analysis. The scores differed significantly between TNC and VNC only for obese patients (BMI_>28_, p < 0.001). Furthermore, the median underestimation was twice as high compared to patients with a lower BMI (BMI_<24_: − 10%, BMI_24−18_: − 8%, BMI_>28_: − 20%). The correlation remained high for all subgroups (r^2^ > 0.9), but the category agreement between TNC and VNC was lowest for BMI_24−28_ at ‘only’ 77%, although the percentage difference was the smallest.

62 patients showed a sinus (HRh_sin_), and 26 showed no sinus heart rhythm (HRh_no_sin_). Both groups showed similar results. Scores differed significantly between TNC and VNC with a median percentage of − 12% and − 13% for HRh_sin_ and HRh_no_sin_. The correlation for HRh_sin_ slightly exceeded the one of HRh_no_sin_ (r^2^ = 0.96 vs 0.91), however, the category agreement was equivalent (79% vs. 81%).

In terms of heart rate, the results began to differ significantly between TNC and VNC from 70 bpm onwards. HR_<60_ and HR_60-69_ showed only small median percentage difference of − 5% and − 6%, a consistently high correlation (r^2^ = 0.93 and 0.98) and an agreement in risk category of 83% and 77%. HR_70-79_ showed an increase in underestimation of scores on VNC with a median difference of − 15% to TNC derived scores. Although the correlation was high with r^2^ of 0.94, the risk agreement was lowest at only 63%. For both heart rate groups above 79 bpm, the median difference reached − 26%, but the first quartile was the most extreme at − 33% for the group of HR_>89_. Correlation and risk category agreement was similarly high for both groups (r^2^ = 0.95 and 0.91, agreement of 89% and 88% for HR_80−89_ and HR_>89_).

Table 3 allows a more detailed examination of risk category agreement and shows the difference between VNC-TNC in the absolute number of patients classified as no, mild, moderate, and severe CAC (see also Fig. 3C). As demonstrated, according to TNC derived scores most (54 out of 88) patients suffered from severe CAC, thereof 10 were misclassified into lower risk category based on VNC scores, 2 into mild and 8 into moderate. Although most of the misclassified patients were in higher BMI groups, more than half showed sinus rhythm and all heart rate groups were represented.

**Table 3:** Agreement in risk category grouped by body mass index, heart rhythm and heart rate

		n	Agreement in Risk Category [n]															
			No (TNC = 0)				Mild (TNC = 1–100)				Moderate (TNC = 101–400)				Severe (TNC > 400)			
			0	1	2	3	− *1*	0	**1**	**2**	− *2*	− *1*	0	**1**	− *3*	− *2*	− *1*	0
Total		88	0	1	0	0	1	10	1	0	1	3	16	1	*0*	2	8	44
BMI [kg/m^2^]	< 24	23	0	0	0	0	1	2	0	0	0	0	8	1	0	0	1	10
	24–28	30	0	1	0	0	0	1	0	0	1	2	5	0	0	1	2	17
	> 28	30	0	0	0	0	0	5	0	0	0	1	3	0	0	1	3	17
Heart rhythm	No sinus	26	0	0	0	0	0	0	1	0	0	0	6	0	0	1	3	15
	Sinus	62	0	1	0	0	1	10	0	0	1	3	10	1	0	1	5	29
Heart rate [bpm]	< 60	12	0	1	0	0	0	1	0	0	0	0	0	0	0	0	1	9
	60–69	22	0	0	0	0	0	1	0	0	0	2	3	1	0	0	2	13
	70–79	19	0	0	0	0	0	1	1	0	1	1	4	0	0	0	4	7
	80–89	18	0	0	0	0	0	5	0	0	0	0	5	0	0	1	1	6
	> 89	17	0	0	0	0	1	2	0	0	0	0	4	0	0	1	0	9

Columns contain the classification into no, mild, moderate and severe calcification according to Agatston scores derived from true non-contrast and the respective difference to the virtual non-contrast-based categorization in range − 3 to 3 (underestimation to overestimation of maximum three categories). The corresponding numbers indicate the classification difference between TNC and VNC, ranging from e.g. − 3 (three categories lower) to + 3 (three categories higher), with 0 representing identical classification

*BMI* body mass index, *n* absolute number of patients, *TNC* true non-contrast, *VNC* pure calcium virtual non-contrast

21 patients had moderate CAC, with VNC showing deviating results in 5 cases, with 1 patient classified as having no, 3 patients as having mild and one patient as having severe CAC. Misclassified patients were overweight or obese, but all showed sinus rhythm and rather low heart rates below 80 bpm.

TNC Agatston scores showed mild CAC in 12 patients and no CAC in 1 patient. Within the mild category, VNC agreed with 10 patients and categorized one as no and one as moderate. The one patient with no CAC on TNC was categorized as mild by VNC. Again, there is no clear trend in the cohort in terms of BMI, heart rhythm or heart rate causing the misclassification.

## Discussion

In this study we performed CACS in a large cohort and evaluated the influence of BMI, heart rhythm and heart rate, on the accuracy of VNC compared to TNC derived calcium scores on PCD-CT data. Main findings of our study are: 1) CACS on VNC underestimates TNC scores but matches CAC severity categorization in 80% of cases; 11% of misclassified cases risk inappropriate treatment. 2) VNC and TNC scores align for non-obese, non-tachycardic patients (< 28 kg/m^2^, < 69 bpm), with differences escalating in obese or tachycardic cases, though risk categorization remains consistent. Coronary CTA has a class 1 indication for the diagnosis of CAD according to current European Society of Cardiology guidelines [22]. In January 2024, the Joint Federal Committee (Gemeinsamer Bundesausschuss) passed a resolution to add the coronary CTA to the statutory health insurance benefit catalog based on conclusive studies [23] which demonstrates the diagnostic value of this imaging technique in the assessment of CAD. The possibility of a reliable quantification of CAC on CTA-derived VNC reconstructions promises a reduction in patient radiation dose and acquisition time to a minimum. Several studies analyzing dual-energy [6, 7, 9, 24, 25] and photon-counting [16, 17] CT data have demonstrated feasibility based on excellent correlation of calcium scores. However, the reproducibility and reliability of calcium quantification based on VNC images in terms of BMI, heart rhythm and heart rate remain unclear.

Overall, an underestimation of CACS was observed, which, in contrast to conventional VNC derived scores, does not require a general correction factor [6, 8]. The effect is probably due to an underestimation of plaque density and volume [6]. However, the agreement in severity categorization was high and the correlation was excellent.

Previously, BMI has been shown to have a negative impact on image quality with respect to CACS in VNC images [10, 26, 27]. Evaluation of the accuracy within BMI groups showed no significant differences in calcium scores for normal weight and mildly obese patients, whereas in obese patients scores differed significantly and the percentage underestimation was doubled. Interestingly, this observation was not reflected regarding high correlation and categorization of CAC severity, which both remained stable even for BMI’s exceeding 28 kg/m^2^. Extreme misinterpretation of scores occurred mainly within one category, especially the severe category, and not across categories. Improved independence of CT values in VNC images from PCD-CT systems from patient BMI has already been demonstrated in other anatomical regions [28, 29].

Cardiac arrhythmias can lead to poor image quality in cardiac imaging [30–32]. One third of the patients included lacked a sinus rhythm. However, the percentage difference, correlation as well as risk categorization agreement comparing TNC and VNC was equivalent for both groups.

Similar to arrhythmia, an increased heart rate can decrease the quality of CT imaging due to myocardial contractility [33]. Therefore, decreasing the heart rate to < 65 bpm has been recommended in cardiac imaging for quite some time [34–36]. This studies subgroup analysis revealed no significant, and in the percentage median minor differences in CAC score for heart rates < 80 bpm. For higher heart rates, the underestimation is extremely enlarged. However, the correlation and the agreement in the risk category was comparable for all categories, except for the middle one, which included heart rates from 70 to 79 bpm, and an agreement of only just two-thirds.

CAC is an independent risk factor for cardiovascular disease [37]. According to guidelines, the quantification can be used to make treatment decisions in patients with elevated cholesterol, especially in those patients in whom statin therapy is still uncertain. Patients with an Agatston score of > 100 have a 7.5% risk of a cardiovascular event within 10 years [37] and therapy with statins is recommended for patients > 40 years [38]. If, on the other hand, the Agatston score is 0, this may indicate a wait-and-see approach to statin therapy in patients at low risk for a cardiovascular events [38]. In this study 6 patients (referring to 7% of the study cohort) were erroneously classified to risk category < 100 according to VNC based Agatston scores and two patients (referring to 2% of the study cohort) showed no measurable CAC on VNC series. If they were considered low or no risk patients, they may have been mistakenly not treated. Vice versa, one patient was erroneously categorized into groups > 100 and one > 0 according to VNC based Agatston scores which would potentially lead to unnecessary therapy.

This study has several limitations. First, although the total number of patients in this study was high, creating subgroups reduces the number within each and therefore the informative values of the results. Larger or even multi-centric studies are needed to confirm the results regarding the influence of patient characteristics on CACS. Second, this study lacks an actual assessment of the differences in clinical decision-making according to the different CACS. However, this would be the consequent next step following the analysis of severity agreement. Third, all results are solely on a quantitative basis analyzing differences, correlation, and severity agreement. Qualitative evaluations and/ or further quantitative measures should be considered in future studies. Fourth, the study cohort consisted of TAVR patients only. This indicates that the patient population is relatively advanced in age and demonstrates an elevated risk of coronary artery calcification. Consequently, severe calcifications were identified and assessed, indicating the necessity for further verification of the conclusions pertaining to low CACS. Fifth, another limitation of the selected TAVR study cohort is the potential for misattribution of aortic or mitral valve calcifications to the coronary arteries, resulting in the measurement of an erroneous value. Sixth, prior research indicates that the new PCD-CT technology provides substantial dose reduction. However, this aspect was not assessed in the current study and warrants investigation in future research efforts.

In conclusion, this study proofed VNC to provide a reliable estimate of TNC-based CACS for non-obese patients (< 28 kg/m^2^) with non-tachycardic sinus rhythm (< 69 bpm) in patients with severe CAD. For obese or tachycardic patients the possibility of underestimation of TNC CACS must be considered for clinical decision making. Further improvements in VNC algorithm might soon allow the substitution of additional TNC scans for CACS.

## Supplementary Information

Below is the link to the electronic supplementary material.

Supplementary file1 (TIF 4946 KB)

## Data Availability

No datasets were generated or analysed during the current study.

## Abbreviations

BMI: Body mass index
CAC(S): Coronary artery calcium (scoring)
CT: Computed tomography
CTA: Computed tomography angiography
CTDI_vol_: Volumetric CT dose index
DLP: Dose length product
PCD: Photon-counting detector
ROI: Region of interest
SSDE: Size-specific dose estimate
TAVR: Transcatheter aortic valve replacement
TNC: True non-contrast
VNC: Virtual non-contrast
